# Combined anti-tumor efficacy of somatostatin fusion protein and vaccinia virus on tumor cells with high expression of somatostatin receptors

**DOI:** 10.1038/s41598-022-21506-8

**Published:** 2022-10-07

**Authors:** Jun Fan, Lili Deng, Ying Peng, Yuedi Ding

**Affiliations:** NHC Key Laboratory of Nuclear Medicine, Jiangsu Key Laboratory of Molecular Nuclear Medicine, Jiangsu Institute of Nuclear Medicine, Wuxi, 214063 Jiangsu China

**Keywords:** Biotechnology, Cell biology, Oncology

## Abstract

Somatostatin, a growth hormone-release inhibiting peptide, exerts antiproliferative and antiangiogenic effects on tumor cells. However, the short half-life of somatostatin limits its application in human therapy, and long-acting somatostatin fusion protein is also limited by its severe terminal degradation. Therefore, oncolytic virus delivery system was introduced to express somatostatin fusion protein and the anti-tumor effects of both somatostatin and oncolytic virus were combined to destroy tumor tissues. Here, a vaccinia VG9/(SST-14)_2_-HSA recombinant was constructed by replacing somatostatin fusion gene into TK locus of attenuated VG9 strain via homologous recombination. Results showed that vaccinia VG9/(SST-14)_2_-HSA possessed a combined anti-tumor effect on sstr-positive tumor cells in vitro. In the tumor burden models, BALB/c mice with complete immunity are most suitable for evaluating tumor regression and immune activation. Complete tumor regression was observed in 3 out of 10 mice treated with vaccinia VG9/TK^−^ or VG9/(SST-14)_2_-HSA, and the survival of all mice in both groups was significantly prolonged. Besides, vaccinia VG9/(SST-14)_2_-HSA is more effective in prolonging survival than VG9/TK^−^. Vaccinia VG9/(SST-14)_2_-HSA exerts a combined anti-tumor efficacy including the oncolytic ability provided by the virus and the anti-tumor effect contributed by (SST-14)_2_-HSA, which is expected to become a potent therapeutic agent for cancer treatment.

## Introduction

Somatostatin (SST) is a growth hormone (GH)-release inhibiting peptide that was originally isolated from ovine hypothalamus in 1973^1^. In the human body, somatostatin is found mainly in the hypothalamus within the central nervous system, and mainly localized in the dorsal root ganglia and sensory nerves within the peripheral nervous system. Outside the central and peripheral nervous system, somatostatin is expressed mainly in the pancreas and gastrointestinal tract, as well as thyroid gland and kidney^2^. Somatostatin acts as a neurotransmitter and neuromodulator in the central nervous system, and acts as a strong inhibitor of growth hormone and thyroid stimulating hormone (TSH) in the anterior pituitary gland. Besides, somatostatin inhibits the secretion of insulin and glucagon, and affects peristalsis and the absorption of nutrients and ions in the gastrointestinal system^3^. Importantly, somatostatin exerts antiproliferative and antiangiogenic effects on cancer cells^4^. Somatostatin exists in two biologically active forms, 14 amino acid (SST-14) and its N-terminally extended 28 amino acid form (SST-28), which is characterized by a cyclic motif containing a disulfide bond between the cysteine residues at positions 3 and 14^5^. Diverse biological activity of natural somatostatin and its analogues is mediated by specific membrane receptors belonging to the family of G protein coupled receptors. Five somatostatin receptor subtypes (sstr1-5) have been defined, whereas sstr2 exists in two forms, sstr2A and sstr2B, which have a different carboxyl terminus^6^. Somatostatin receptors are expressed in both normal and pathological tissues and often over-expressed in the tumoral tissues^3^.

Due to enzymatic degradation, natural somatostatin has a very short serum half-life of 1–3 min^5,7^. To improve the pharmacokinetic profile, more potent and long-acting analogs of somatostatin were synthesized and commercialized, including octreotide, lanreotide, vapreotide and pasireotide (SOM230). However, the binding affinity of the analogs to the somatostatin receptor subtypes is different from natural somatostatin. Natural somatostatin has universal high nanomolar affinity to all five receptor subtypes (sstr1-5), while octreotide and lanreotide only bind with high affinity to sstr2 and sstr5^7,8^. Different binding affinities to receptor subtypes may lead to different biological and clinical activities, so the natural somatostatin seems to be the best candidate ignoring its short half-life. Therefore, Human serum albumin (HSA) fusion technology was applied to extend the circulatory half-life of somatostatin by fusing the natural somatostatin with a full-length HSA molecule.

Ding et al. have constructed a number of human somatostatin fusion proteins expressed by the Pichia pastoris system, that is, two or three copies of SST-14 or SST-28 were fused to the C or N terminus of the HSA molecule, and found (SST-14)_2_-HSA and (SST-28)_2_-HSA showed better bioactivity in inhibiting GH secretion^9,10^. HSA is the most prevalent natural blood protein in the human circulatory system with a long half-life of 19 days, which makes it ideal for conjugation with small therapeutic proteins to create long-acting forms of fusion proteins^11^. However, there is also some limitations about this technology, such as low expression levels of somatostatin fusion proteins and protein degradation at N or C terminus occurred in the fermentation and purification process. Hence, we introduced a vaccinia virus delivery system, which expresses somatostatin fusion protein for cancer therapy, and vaccinia virus itself also has an oncolytic effect. This technology avoids the terminal degradation of somatostatin protein and the complicated purification process, while the tumor tropism of vaccinia virus can transport the fusion protein to the tumor region and exert a better anti-tumor effect.

Vaccinia virus, a member of the Orthopoxvirus genus, was used to vaccinate against smallpox from the eighteenth century to the twentieth century until smallpox was eradicated in 1977^12^. Then, vaccinia virus was applied as an agent for oncolytic viral therapy due to several advantages. First, vaccinia virus has been used as a live vaccine in the smallpox vaccination program for over 200 million people worldwide, making it a safe and attractive oncolytic agent. Besides, the large dsDNA genome of vaccinia virus makes it well suited to transgene delivery by insertion of exogenous therapeutic genes, and cytoplasmic replication reduces the chance of viral DNA recombination into the host genome^13,14^. Vaccinia virus is capable of infecting a wide variety of cells and naturally selective for tumors^13^. Further selectivity was achieved through the deletion of the viral genes that are essential for virus replication in normal cells, such as thymidine kinase (TK) and vaccinia growth factor (VGF)^13,15^. TK-deficient virus requires thymidine triphosphate from the nucleotide pool for DNA synthesis, while thymidine triphosphate synthesized by TK is only present in dividing cells. This leads to preferential viral replication in dividing cells, thereby improving the tumor specificity of vaccinia virus^15,16^. VGF is secreted in the early stage of viral infection and acts as a mitogen to trigger non-infected cells for subsequent infection. VGF is required for efficient viral replication, and the absence of VGF reduces the virus virulence in resting cells and increases the LD_50_ of intracranial vaccinia^15,17,18^.

In previous study, we have constructed a TK deleted vaccinia virus Tian Tan strain Guang9 (VG9), which was historically used for the vaccination of smallpox in China. Tumor selectivity of TK-deficient VG9 (VG9/TK^−^) was validated by replacing TK gene with luciferase gene and monitoring by in vivo bioluminescence imaging. It was confirmed that VG9/TK^−^ is tumor tropism in both immunocompromised and immunocompetent mice, and by intratumoral or intraperitoneal or intravenous administration^19,20^. Due to the safety and efficacy of the oncolytic virus VG9/TK^−^, we applied this promising transgenic vector to express somatostatin fusion protein, and the therapeutic effects of both somatostatin and oncolytic virus were combined to destroy tumor tissues.

## Results

### Construction and characterization of vaccinia VG9/(SST-14)_2_-HSA

**Figure 1:**
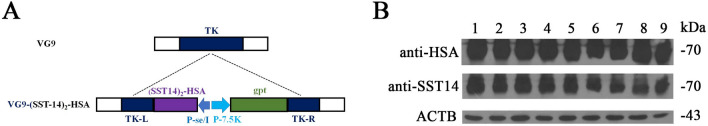
Characterization of vaccinia VG9/(SST-14)_2_-HSA. (**A**) Schematic illustration of VG9/(SST-14)_2_-HSA construction. Somatostatin fusion protein (SST-14)_2_-HSA expression cassette was inserted into TK locus of VG9 strain via homologous recombination. In the expression cassette, gpt gene is driven by the promoter P-7.5 K in cis direction while SST14-HSA is driven by the reverse promoter P-se/I. (**B**) Western blot analysis of the immunogenicity of HSA and somatostatin in different vaccinia VG9/(SST-14)_2_-HSA recombinants. The blots were cut prior to hybridization with the antibody to control protein of β-actin during blotting.

The recombinant vaccinia VG9/(SST-14)_2_-HSA was generated by inserting human somatostatin fusion gene (SST-14)_2_-HSA into TK locus of attenuated VG9 strain via homologous recombination (Fig. 1A). To confirm the expression of (SST-14)_2_-HSA, western blot was applied to validate the immunogenicity of somatostatin and HSA in the fusion protein. As shown in Fig. 1B, all 9 recombinants expressed the target gene and possessed the immunogenicity of both somatostatin and HSA, indicating that vaccinia VG9/(SST-14)_2_-HSA has been successfully constructed. The recombinants No.1 with the highest expression level was selected for further experiment.

### Expression of somatostatin receptors (sstrs) in tumor cells

**Figure 2:**
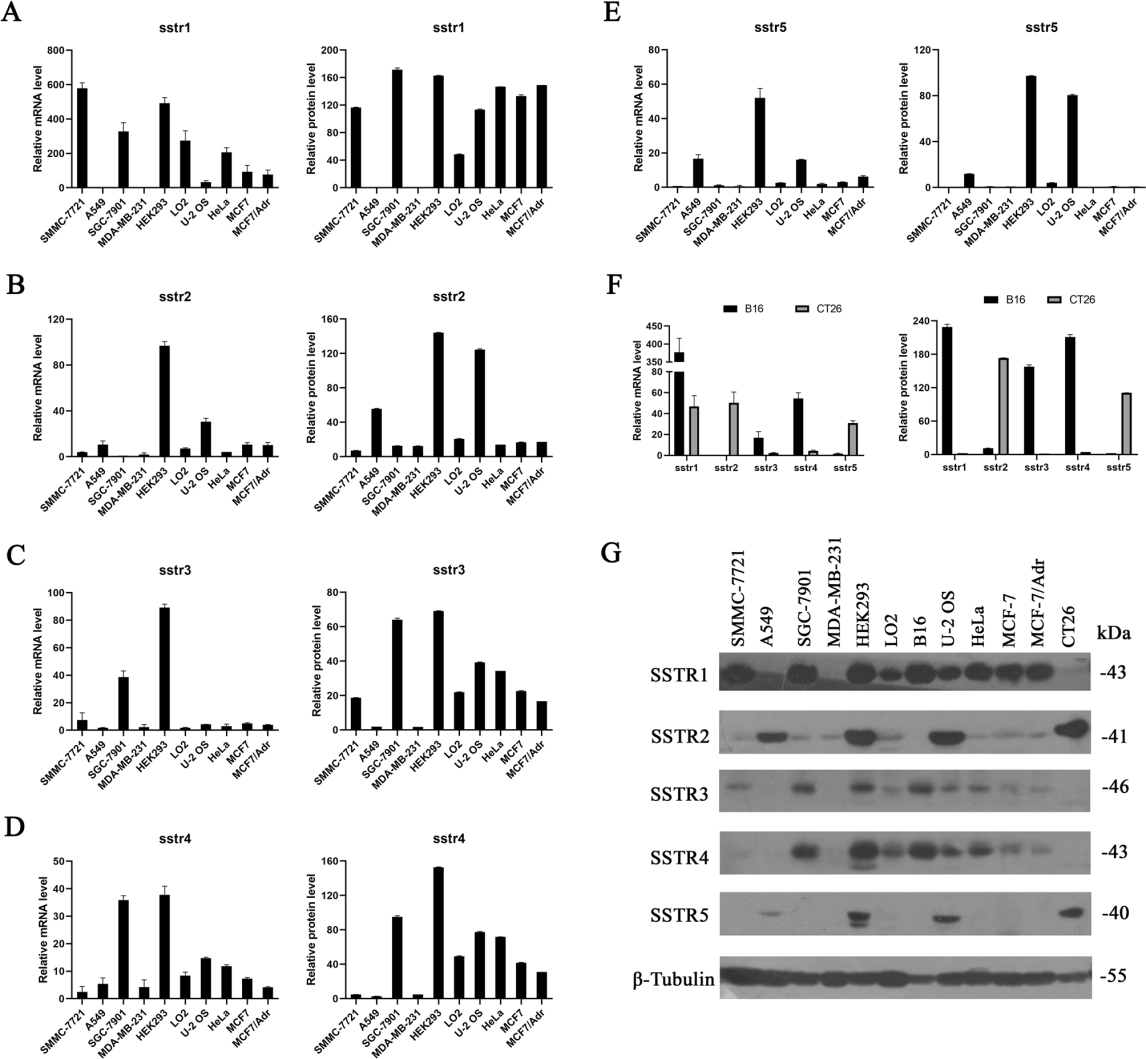
Screening of somatostatin receptors (sstrs) expressed in human and murine cell lines. (A-E) Expression levels of sstr1 (**A**), sstr2 (**B**), sstr3 (**C**), sstr4 (**D**) and sstr5 (**E**) in normal and tumor human cell lines. mRNA levels analyzed by real-time PCR are shown on the left, and protein levels quantified by western blot are shown on the right. Each bar represents the means ± SD (n = 3). (**F**) Relative mRNA levels (left) and semi-quantitative protein levels (right) of sstr1-5 expressed in murine cell lines. (**G**)Western blot analysis of sstr1-5 in normal and tumor cell lines.

SST-14 is chosen as the therapeutic protein because natural somatostatin has universal high nanomolar affinity to all five receptor subtypes (sstr1-5). Therefore, expression of the corresponding somatostatin receptors in target cells is the basis for the biological activity of SST-14, and screening the expression of sstrs in tumor cells is the first thing to do. Here, quantitative real-time PCR was applied to determine the gene expression of sstrs, while western blot was used to confirm the protein levels of sstrs. Figure 2 revealed that the relative mRNA and protein levels of sstr1-5 in various cell types were consistent. HEK-293, an embryonic kidney cell line, expresses all 5 sstrs at high levels, while the other normal hepatic cells LO2, only weakly expresses sstr1 and sstr4. In tumor cell, bone osteosarcoma U-2 OS expresses all 5 sstrs at moderate levels. Murine melanoma B16 and human gastric carcinoma SGC-7901 express high levels of sstr1, sstr3 and sstr4, while murine colon carcinoma CT26 and human lung carcinoma A549 highly express sstr2 and sstr5. None of any sstr was expressed in human breast adenocarcinoma MDA-MB-231, while only sstr1 was expressed in the remaining tumor cells.

### Oncolytic potency of vaccinia VG9/(SST-14)_2_-HSA in vitro

**Figure 3:**
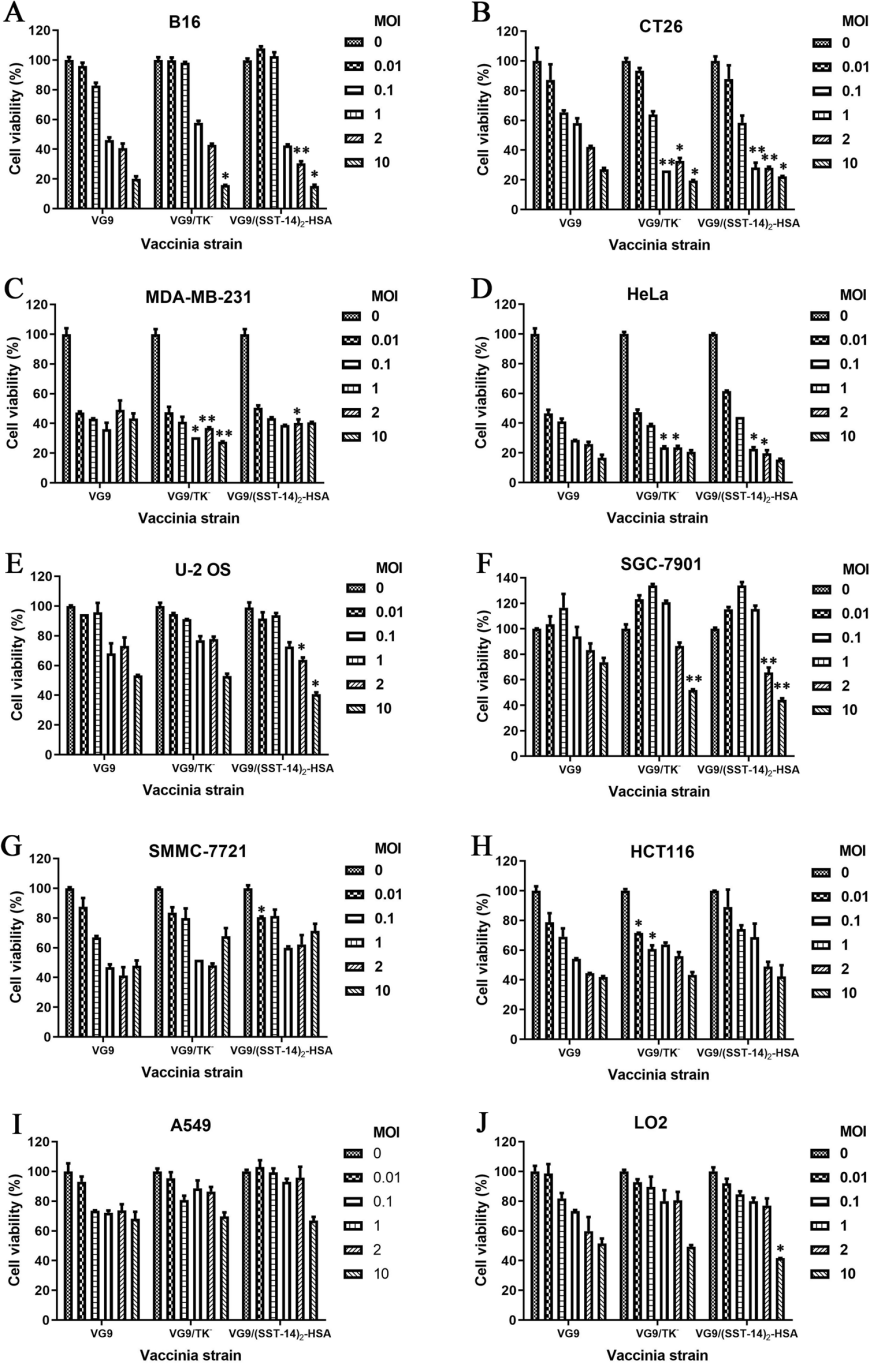
In vitro oncolytic potency of vaccinia VG9/(SST-14)_2_-HSA. Murine cell lines of B16 (**A**) and CT26 (**B**), and human cell lines of MDA-MB-231 (**C**) and HeLa (**D**), U-2 OS (**E**) and SGC-7901 (**F**), SMMC-7721 (**G**), HCT116 (**H**), A549 (**I**) and normal cell line LO2 (**J**) were infected with increasing titers of VG9, VG9/TK^−^ and VG9/(SST-14)_2_-HSA (MOI = 0, 0.01, 0.1, 1, 2 and 10) for 72 h. Cell viability was measured by MTT assay. Each bar represents the means ± SD (n = 3). *p < 0.05, **p < 0.01. *p* values represent the significant differences in cell viability across different vaccinia strain.

To verify the oncolytic potency of VG9/(SST-14)_2_-HSA on different tumor cells, various cells were infected with increasing titers of VG9, VG9/TK^−^ and VG9/(SST-14)_2_-HSA. As shown in Fig. 3, tumor cells including B16, CT26, MDA-MB-231 and HeLa were sensitive to all three viruses, and no significant difference was observed in the high oncolytic activity of the viruses. Whereas, the oncolytic activity of vaccinia viruses was not high enough in other tumor cells including U-2 OS, SGC-7901, SMMC-7721, HCT116, A549, indicating these cells were more resistant to the viruses. LO2, as the non-tumor hepatic cell line, was resistant to viruses as well. Among these viruses, wild-type vaccinia VG9 has the highest oncolytic ability on cells. TK-deleted vaccinia VG9/TK^−^ that does not express any exogenous gene has relatively low oncolytic ability compared to VG9. Vaccinia VG9/(SST-14)_2_-HSA possesses even weaker oncolytic ability than VG9/TK^−^ due to the insertion of exogenous gene, however, the expressed (SST-14)_2_-HSA can improve the oncolytic ability of sstrs-positive tumor cells through the interaction with sstrs. Tumor cells B16, CT26, U-2 OS and SGC-7901 that expressing 2–5 subtypes of sstrs, were more sensitive to vaccinia VG9/(SST-14)_2_-HSA than VG9/TK^−^ at high MOI, which proved that vaccinia virus and (SST-14)_2_-HSA possess a combined oncolytic effect. As a result, U-2 OS, B16 and CT26, which originate from human, C57BL/6 and BALB/c mice, respectively, were used to verify the oncolytic ability of vaccinia virus in vivo.

### Anti-tumor efficacy of vaccinia VG9/(SST-14)_2_-HSA in vivo

**Figure 4:**
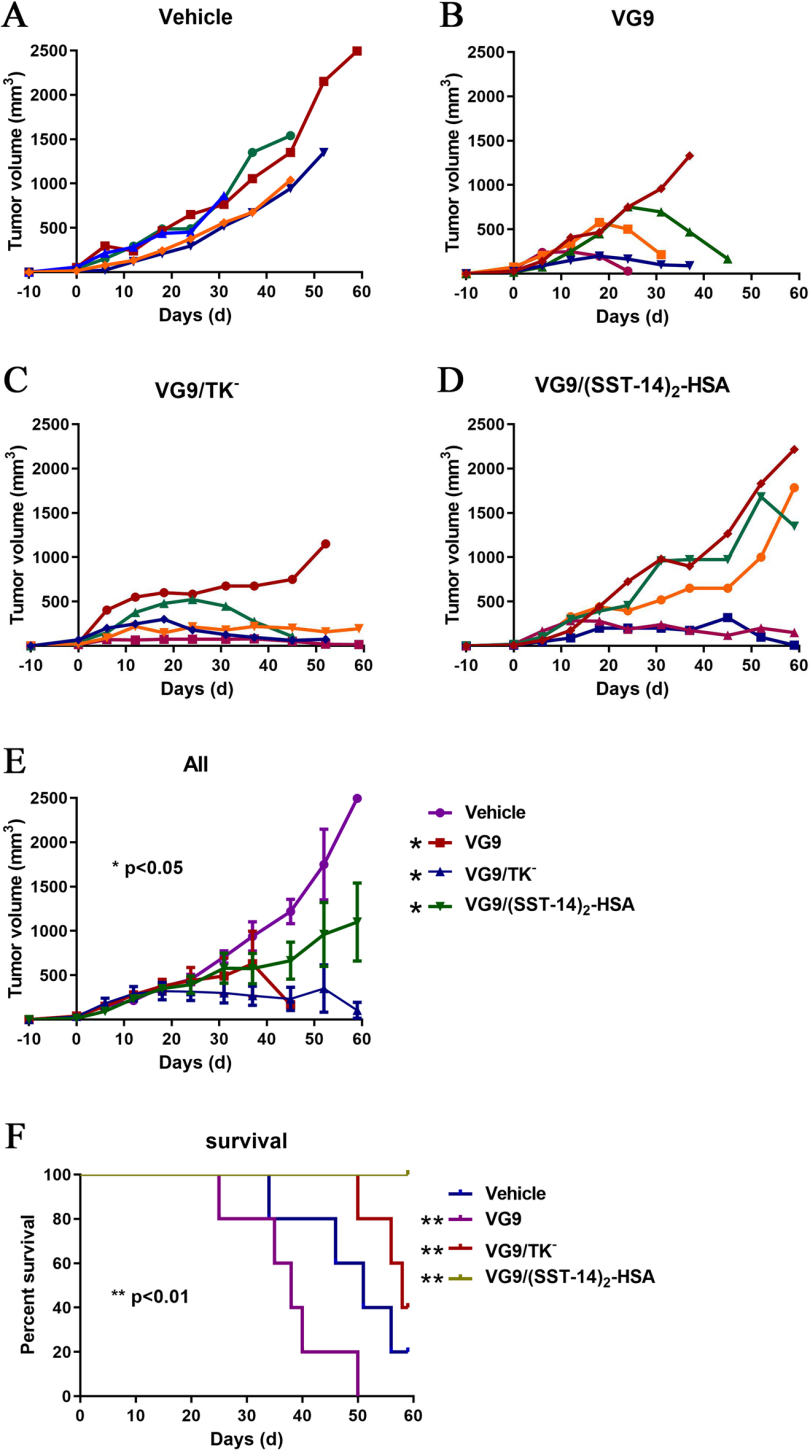
Anti-tumor efficacy of vaccinia VG9/(SST-14)_2_-HSA in nude mice bearing U-2 OS tumor cells. (**A**–**D**) Tumor volumes in nude mice treated with PBS (**A**), VG9 (**B**), VG9/TK^−^ (**C**), VG9/(SST-14)_2_-HSA (**D**). (**E**) Average tumor volumes of mice received virotherapy (n = 5). *p < 0.05. (**F**) Kaplan-Meier survival curves of tumor-bearing nude mice treated with vaccinia viruses. **p < 0.01.

Nude mice model established by human bone osteosarcoma U-2 OS was applied to investigate the anti-tumor efficacy of vaccinia VG9/(SST-14)_2_-HSA. Vaccinia VG9, VG9/TK^−^ and VG9/(SST-14)_2_-HSA were intratumorally injected into tumor-bearing nude mice, then tumor volume and survival status were monitored for up to 60 days. As shown in Fig. 4, all three vaccinia VG9, VG9/TK^−^ and VG9/(SST-14)_2_-HSA could effectively suppress tumor progression in nude mice, although the tumor remission ability of vaccinia VG9/(SST-14)_2_-HSA was weak. However, the survival curve showed a different situation. Vaccinia VG9 possessed good tumor regression ability, but exerted the worst survival rate. Tumor-bearing nude mice treated with VG9 died even earlier than control mice, indicating the high cytotoxicity of the wild strain. Whereas, when the TK gene is deleted, VG9/TK^−^ displayed better survival ability and stronger anti-tumor effect. In addition, insertion of exogenous therapeutic gene further improved the survival of tumor-bearing mice. All mice survived after intratumoral injection of VG9/(SST-14)_2_-HSA, although the effect of tumor suppression was slightly weak.

**Figure 5:**
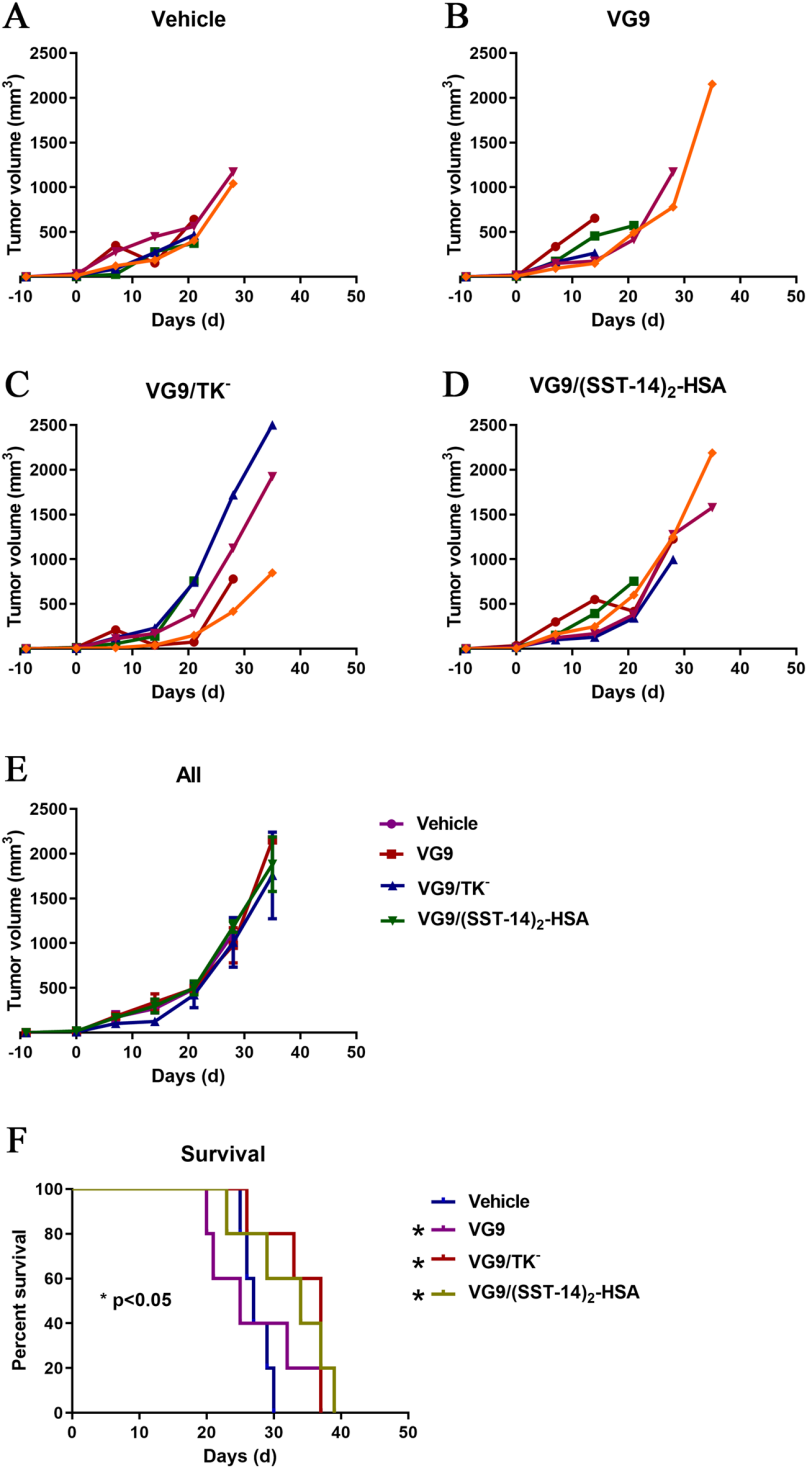
Anti-tumor efficacy of vaccinia VG9/(SST-14)_2_-HSA in C57BL/6 mice bearing B16 tumor cells. (**A**–**D**) Tumor volumes in C57BL/6 mice treated with PBS (**A**), VG9 (**B**), VG9/TK^−^ (**C**), VG9/(SST-14)_2_-HSA (**D**). (**E**) Average tumor volumes of mice received virotherapy (n = 5). (**F**) Kaplan-Meier survival curves of tumor-bearing C57BL/6 mice treated with vaccinia viruses. *p < 0.05.

As an oncolytic virus, vaccinia can activate immune response of the host. Therefore, the immunocompetent C57BL/6 and BALB/c mice were also investigated to consider the influence of host immune system on the anti-tumor effect of vaccinia virus. Figure 5 showed the tumor volume and survival status of C57BL/6 mice bearing murine melanoma B16. Under the therapeutic of vaccinia VG9, VG9/TK^−^ and VG9/(SST-14)_2_-HSA, tumors were not suppressed as expected due to the prolonged survival period. Among these viruses, the wild strain VG9 displayed high toxicity, and most mice died even earlier than control mice. Whereas, the survival of mice treated with VG9/TK^−^ or VG9/(SST-14)_2_-HSA was significantly prolonged, although tumor regression was not observed in the mice. Results proved the therapeutic effect of the viruses VG9/TK^−^ and VG9/(SST-14)_2_. As shown in Fig. 5E, all mice including the control group died within 40 days after virus injection, suggesting that the immune system of C57BL/6 mice played an important role in mouse survival.

**Figure 6:**
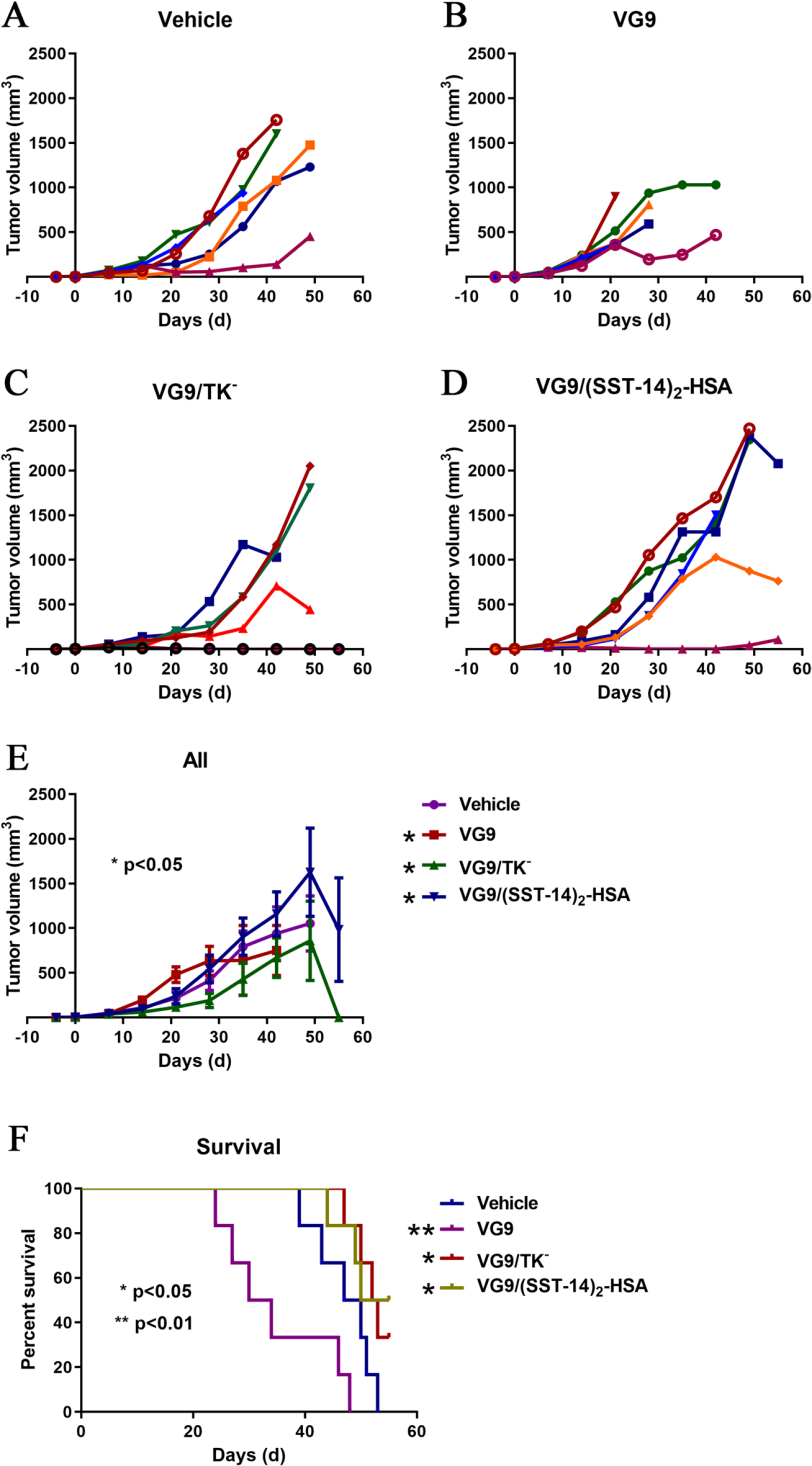
Anti-tumor efficacy of vaccinia VG9/(SST-14)_2_-HSA in BALB/c mice bearing CT26 tumor cells. (**A**–**D**) Tumor volumes in BALB/c mice treated with PBS (**A**), VG9 (**B**), VG9/TK^−^ (**C**), VG9/(SST-14)_2_-HSA (**D**). (**E**) Average tumor volumes of mice received virotherapy (n = 6). *p < 0.05. (F) Kaplan-Meier survival curves of tumor-bearing BALB/c mice treated with vaccinia viruses. *p < 0.05, **p < 0.01.

Similarly, Fig. 6 showed the therapeutic effect of vaccinia viruses on BALB/c mice bearing murine colon carcinoma CT26. In general, tumor-bearing BALB/c mice survived much longer than C57BL/6 mice, and a total of 5 mice survived over 60 days after virus injection. Same as C57BL/6 mice, BALB/c mice treated with VG9/TK^−^ or VG9/(SST-14)_2_-HSA showed much better survival status. There were 2 mice treated with VG9/TK^−^ and 3 mice treated with VG9/(SST-14)_2_-HSA survived finally. In contrast, the survival status of BALB/c mice treated with VG9 was poor, and all mice died at least 7 days earlier than control mice. Similarly, most BALB/c mice treated with VG9/TK^−^ or VG9/(SST-14)_2_-HSA failed to inhibit tumor progression. Whereas, complete tumor regression was observed in 2 mice treated with VG9/TK^−^ and 1 mouse treated with VG9/(SST-14)_2_-HSA. These results further demonstrated the therapeutic effect of vaccinia VG9/TK^−^ and VG9/(SST-14)_2_-HSA, and VG9/(SST-14)_2_-HSA is more potent in prolonging survival. Meanwhile, BALB/c mice are more suitable as tumor burden models to evaluate the role of viruses in tumor regression and immune activation.

## Discussion

The biological activity of somatostatin is mediated directly by somatostatin receptors^21,22^. Unlike its synthetic analogs, natural somatostatin binds to all 5 sstrs with equally high affinity^23^. Therefore, over-expression of sstr1-5 in tumors is the molecular basis for the therapeutic application of somatostatin. In order to exert the combined therapeutic effect of vaccinia VG9/(SST-14)_2_-HSA, besides the oncolytic effects of vaccinia, tumors must be sstr-positive to activate the anti-tumor effect of the expressed protein (SST-14)_2_-HSA. It is reported that all sstrs are expressed in a variety of normal human tissues, primary located in the central and peripheral nervous systems, gastrointestinal tract and endocrine organs^24^. In addition, sstrs are highly expressed in pituitary adenomas, gastroenteropancreatic and neuroendocrine tumors, and are expressed at varying degrees in solid organ tumors, such as prostate, breast, ovarian, thyroid, and hepatocellular carcinoma, as well as melanoma and gastrointestinal tumor tissues^24^. Therefore, human or murine carcinomas of lung, hepatic and gastric, osteosarcoma, melanoma, cervix, colorectal, mammary, as well as normal embryonic kidney and hepatic cells were investigated and the expression of sstrs was verified. Not all receptors are expressed in these cells except for HEK-293 and U-2 OS, and no receptor is expressed in MDA-MB-231. Besides A549 and CT26 which only express sstr2 and sstr5, other cells express sstr1 at least, and some of them also express sstr3 and sstr4 at the same time. We found that the expression of sstr2 and sstr5 is complementary to the expression of sstr1, sstr3 and sstr4, and only one of them can be expressed unless all sstr1-5 are expressed in the cell. As a result, tumor cells with different sstrs expression were chosen for virotherapy assay, including U-2 OS which expresses all sstr1-5, B16 that highly expresses sstr1, sstr3 and sstr4, and CT26 expressing high sstr2 and sstr5.

Sstrs seems to be a suitable biomarker for targeted therapy of vaccinia (SST-14)_2_-HSA, whereas, the oncolytic efficacy of the vaccinia virus is another indicator of targeted therapy. Thus, in vitro oncolytic potency of vaccinia VG9, VG9/TK^−^ and VG9/(SST-14)_2_-HSA on carcinomas of lung, hepatic and gastric, osteosarcoma, melanoma, cervix, colorectal, mammary, as well as normal hepatic cells were compared. We found that human breast adenocarcinoma MDA-MB-231and human cervix adenocarcinoma HeLa were extremely sensitive to vaccinia virus, while human lung carcinoma A549 and human bone osteosarcoma U-2 OS, as well as the normal hepatic cell LO2, were more resistant to viruses. Results also showed that VG9, as a wild-type vaccinia virus, displayed excellent oncolytic ability. Deletion of TK gene without exogenous gene insertion moderately decreased its oncolytic ability, while insertion of exogenous gene into the TK locus further reduced its oncolytic ability. However, with the anti-tumor effect of (SST-14)_2_-HSA, the combined cytotoxicity of VG9/(SST-14)_2_-HSA was higher than VG9/TK^−^. The conclusion was confirmed by the results of sstr-positive cells infected by VG9/(SST-14)_2_-HSA with MOI of 1, 2 and 10. Specifically, the sstr-negative tumor cell MDA-MB-231 did not show any combined oncolytic ability under the effect of VG9/(SST-14)_2_-HSA at high MOIs. Nevertheless, selected sstr-positive tumor cells, such as U-2 OS, B16 and CT26, displayed excellent combined therapeutic effect.

In order to verify the therapeutic potency of vaccinia VG9/(SST-14)_2_-HSA in the preclinical stage, human bone osteosarcoma U-2 OS cell line expressing all sstr1-5 was applied to establish a nude mice tumor model. Potent therapeutic effect of vaccinia VG9/(SST-14)_2_-HSA was observed in tumor-bearing nude mice, especially in terms of prolonging survival. Besides direct oncolysis, vaccinia virus can induce systemic anti-tumor immunity and lead to tumor clearance^25^. Therefore, the host immune system is also a factor that should be considered in the therapeutic process. As a result, immunocompetent C57BL/6 and BALB/c mice were used to investigate the therapeutic efficacy of vaccinia VG9/(SST-14)_2_-HSA in the presence of host immune system. We found the survival of tumor-bearing C57BL/6 mice was much lower than that of BALB/c mice, both in control mice and virus treated mice. We have reported earlier that the immune system of BALB/c is more complete than C57BL/6 mice, and they can clear the tumor and virus more effectively by the generation of anti-tumor and anti-viral antibodies^16,20^. Therefore, the activation of the host immune system can improve the clearance of tumors and promote anti-tumor efficacy, and BALB/c mice with complete immunity can reveal the therapeutic efficacy of oncolytic virus more effectively. The toxicities observed in the immunocompetent mouse models may be due to an adverse immune response to the therapy given that this toxicity was not observed in the immunocompromised setting.

In the BALB/c tumor model bearing sstr-positive tumor cell CT26, the anti-tumor effects of wild vaccinia VG9, TK-deficient vaccinia VG9/TK^−^ and protein expression vaccinia VG9/(SST-14)_2_-HSA were compared. Results revealed that vaccinia VG9 was highly toxic, because the survival of VG9 treated mice was remarkable shorter than that of control mice. However, vaccinia VG9/TK^−^ treated mice showed excellent tumor remission and survival, indicating that deletion of TK gene minimized the toxicity of VG9. Moreover, vaccinia VG9/(SST-14)_2_-HSA exerted a combined effect including the oncolytic ability provided by the virus and the anti-tumor effect contributed by exogenous (SST-14)_2_-HSA, and exhibited a higher survival rate than VG9/TK^−^. Therefore, vaccinia VG9/(SST-14)_2_-HSA is potent in cancer treatment as a viral therapeutic agent.

## Conclusions

The vaccinia VG9/(SST-14)_2_-HSA, a TK-deficient VG9 strain expressing somatostatin fusion protein was constructed, and its cancer cell inhibition efficacy was evaluated. Sstr-positive cancer cells were chosen as the therapeutic target of vaccinia VG9/(SST-14)_2_-HSA, because the anti-tumor effect of (SST-14)_2_-HSA must be activated through the interaction with sstrs. In vitro cytotoxicity assay between sstr-positive and sstr-negative cells proved the combined anti-tumor effect of vaccinia VG9/(SST-14)_2_-HSA on sstr-positive tumor cells. In the evaluation of the anti-tumor effects of vaccinia viruses on both immunodeficient and immunocompetent tumor burden mice, it was found that vaccinia VG9/(SST-14)_2_-HSA showed excellent therapeutic efficacy in all mice models, especially in terms of prolonging survival. Overall, vaccinia VG9/(SST-14)_2_-HSA, which has both the oncolytic effect of the virus and the anti-tumor effect of (SST-14)_2_-HSA, is a promising anti-tumor agent for virotherapy.

## Materials and methods

### Cell lines

Cell lines, including Vero and BSC-40 (both African green monkey kidney epithelial cells), HEK-293 (human embryonic kidney cells), LO2 (human non-tumor hepatic cells), B16 (murine melanoma), CT26 (murine colon carcinoma), HCT116 (human colorectal carcinoma), A549 (human lung carcinoma), U-2 OS (human bone osteosarcoma), HeLa (human cervix adenocarcinoma), SMMC-7721 (human hepatocarcinoma), SGC-7901 (human gastric carcinoma), MDA-MB-231 (human breast adenocarcinoma), and MCF7 (human mammary adenocarcinoma) were obtained from the Cell Library of Biochemistry and Cell Biology, CAS (Shanghai, China) or the American Type Culture Collection (ATCC, VA, USA), while MCF7/Adr (adriamycin-resistant MCF7 cells) cell line was purchased from Shanghai Bogoo Biotech, China. All cells were cultured in RPMI-1640 or DMEM medium supplemented with 10% heat-inactivated fetal bovine serum, 100 U/ml penicillin, and 100 μg/ml streptomycin. All cell lines were cultured at 37 °C in a humidified 5% CO_2_ incubator.

### Animals

C57BL/6, BALB/c and BALB/c-nu mice (5–6 weeks old) were purchased from Cavens Laboratory Animal (Changzhou, China). All animals were housed under standardized pathogen-free conditions with controlled temperature and humidity and a 12–12 h day-night light cycle. The animal experiment was approved by the Institutional Animal Care and Use Committees (IACUC) of Jiangsu Institute of Nuclear Medicine (JSINM2010007). We confirm that all experiments were performed in accordance with relevant guidelines and regulations, as well as in compliance with the ARRIVE guidelines (https://arriveguidelines.org).

### Construction of vaccinia VG9/(SST-14)_2_-HSA

DNA sequence of HSA refers to GenBank accession No. NM_000477.7 (mat_peptide 114.0.1868), and the sequence of somatostatin refers to GenBank accession No. NM_001048.4 (mat_peptide 410.0.451).

The fusion gene (SST-14)_2_-HSA was amplified from the plasmid pPIC9K-(SST-14)_2_-HSA^9^ with primers P1: 5’-TGTCGACATGGCTGGCTGCAAGAATTTCTTCTG-3’ and P2: 5’-GCGGGAATTCTTATAAGCCTAAGGCAGCTTGAC-3’. After amplification, the obtained DNA was inserted into the *Sal*I and *EcoR*I restriction sites of the pCB plasmid, making it under the control of the vaccinia synthetic early/late promoter (Fig. 1A).

For vaccinia virus transfection, HEK-293 cells were first infected with vaccinia VG9 strain (National Institutes for Food and Drug Control, Beijing, China) for 2 h. Then, the recombinant pCB-(SST-14)_2_-HSA plasmid was transfected into the infected HEK-293 cells with Effectene® Transfection Reagent (Qiagen, Germany), so that the (SST-14)_2_-HSA gene could replace the TK gene of the VG9 strain through homologous recombination of its TK flanking sequences. After that, the recombinants were selected by xanthine-guanine phosphoribosyltransferase (XGPRT) selection in Vero cells in the presence of mycophenolic acid, xanthine and hypoxanthine. The recombinants VG9/(SST-14)_2_-HSA were plaqued for 6 rounds of selection to ensure its purity. The selected recombinants were propagated in BSC-40 cells and purified by centrifugation through a sucrose gradient. Plaque assay was performed on Vero cells to determine the titer of purified vaccinia VG9/(SST-14)_2_-HSA, which is expressed in plaque-forming units (PFU)/ml.

### Characterization of vaccinia VG9/(SST-14)_2_-HSA by western blot

After 6 rounds of plaqued selection, 9 selected vaccinia VG9/(SST-14)_2_-HSA were propagated in BSC-40 cells. Then, the infected cells and supernatant were harvested and lysed through 3 freeze-thaw cycles. The lysates of vaccinia were separated by 10% SDS-PAGE gel and transferred to a PVDF membrane. The membrane was blocked with 3% BSA and incubated with primary antibodies against HSA (ab83465, Abcam, London, UK), somatostatin (ab53165, Abcam) and β-actin (Santa Cruz, CA, USA). Finally, corresponding HRP-conjugated anti-IgG secondary antibodies were incubated and the blots were developed by an ECL luminol reagent (Santa Cruz).

### Screening of somatostatin receptors (sstrs) expression in cells

Normal cells HEK-293, LO2 and tumor cells B16, CT26, A549, U-2 OS, HeLa, SMMC-7721, SGC-7901, MDA-MB-231, MCF7 and MCF7/Adr were seeded in 6-well plates and grown to complete confluence. Total RNA of the cells was extracted by RNAiso Plus Reagent (Takara, Dalian, China) according to the manufacturer’s instructions. cDNA was generated from 3 μg of total RNA with High Capacity cDNA Reverse Transcription Kit (Applied Biosystems, Carlsbad, CA, USA) and the amplification of cDNA with TaqMan probes was performed using TaqMan 2* Universal PCR Master Mix (Applied Biosystems) by an ABI PRISM 7500 Real-Time PCR System (Applied Biosystems). Primers and probes for human sstr1-5 detection in human cell lines and mouse sstr1-5 detection in mouse cell lines were designed and synthesized by GenePharma (Shanghai, China). Human and mouse gapdh genes were used as the normalization controls. The PCR process was set to run at 95 °C for 30 s, 40 cycles of 95 °C for 5 s, 55 °C for 30 s.

The protein of the above-mentioned cells was also obtained by lysing cells in cell lysis buffer (Beyotime, Shanghai, China), and separated by 10% SDS-PAGE. Western blot with antibodies sstr1 (sc-11604), sstr2 (sc-25676), sstr3 (sc-25677), sstr4 (sc-25678), sstr 5 (sc-25679), and β Tubulin (sc-55529) (Santa Cruz, CA, USA) was performed to screen the expression of sstrs in tumor and normal cells.

### Cytotoxicity assay

Normal cells LO2 and tumor cells U-2 OS, B16, CT26, HCT116, HeLa, MDA-MB-231, SMMC-7721, SGC-7901 and A549 were seeded into 96-well plates at the density of 1 × 10^4^ cells/well and grown overnight. Discard the medium and replace with fresh medium containing vaccinia viruses (VG9, VG9/TK^−^, VG9/(SST-14)_2_-HSA) at different multiplicity of infections (MOIs) (0, 0.01, 0.1, 1, 2 and 10). After 72 h of infection, 10 μl of 3-(4,5-dimethylthiazol-2-yl)-2,5-diphenyltetrazolium bromide (MTT) solution was added to each well, and the cells were incubated at 37 °C for 4 h. Discard the supernatant, add 150 μl of dimethylsulfoxide and shake the plate at room temperature for 10 min to completely solubilize the formazan crystals. Then, read the plate at 490 nm on a SpectraMax M5 Multi-Mode Microplate Reader (Molecular Devices, CA, USA). The relative cell viability (%) is calculated by the formula: (absorbance of experimental samples—background absorbance) / (absorbance of untreated Controls—background absorbance) × 100%.

### Animal experiments

Immunodeficient BALB/c-nu mice and immunocompetent C57BL/6 and BALB/c mice were used to establish carcinoma tumor models by implanting corresponding tumor cells. Briefly, 2 × 10^6^ cells of U-2 OS, B16 and CT26 were subcutaneously implanted into the left oxter of BALB/c-nu, C57BL/6 and BALB/c mice, respectively. When the tumors reached 5–8 mm in diameter after 4–10 days of tumor growth, 1 × 10^7^ PFU of purified vaccinia virus (VG9, VG9/TK^−^ and VG9/(SST-14)_2_-HSA) were injected into the mice intratumorally. Tumor growth was monitored weekly and tumor volume (mm^3^) was calculated according to the formula (L × H × W)/2, Where L represents the length, W represents the width and H represents the height of the tumor in millimeters. 60 days after the virus injection, or when tumor volume reached 2,000 mm^3^, or the percentage of body weight loss exceed 20%, mice were euthanized by introducing 100% carbon dioxide (CO_2_) at a flow rate of 4 l/min for 15 min, and the survival curves were plotted.

### Statistical analysis

Survival data was plotted by the Kaplan-Meier method, and differences between curves were assessed using the log-rank test. Statistical analysis was performed by GraphPad Prism 8.0 software (GraphPad, San Diego, CA, USA).

### Ethics approval and consent to participate

All animal procedures were performed in accordance with the Laboratory Animal-Guideline of welfare ethical review of Chinese Institutional Animal Care and Use Committee (IACUC).

## Supplementary Information

Supplementary Information 1.

Supplementary Information 2.

## Data Availability

DNA and protein sequences of somatostatin fusion protein generated during the current study are available in the GenBank repository (https://www.ncbi.nlm.nih.gov/nuccore) under the Accession No. ON454072.
