# Supplementary material for: Combined anti-tumor efficacy of somatostatin fusion protein and vaccinia virus on tumor cells with high expression of somatostatin receptors
Source: Sci Rep. 2022 Oct 7;12:16885. doi: 10.1038/s41598-022-21506-8 (PMC9547013; doi:10.1038/s41598-022-21506-8)
Supplement: Supplementary file 1 — Supplementary Information 1. [file 41598_2022_21506_MOESM1_ESM.docx]

DNA and protein sequences of somatostatin fusion protein (SST-14)_2_-HSA

FASTA sequences

>Seq1 [organism=Homo sapiens] fusion protein of human somatostatin (SST) doublet and human albumin mRNA, complete cds

atggctggctgcaagaatttcttctggaagactttcacatcctgtgctggctgcaagaatttcttctggaagactttcacatcctgtgatgcacacaagagtgaggttgctcatcgatttaaagatttgggagaagaaaatttcaaagccttggtgttgattgcctttgctcagtatcttcagcagtgtccatttgaagatcatgtaaaattagtgaatgaagtaactgaatttgcaaaaacatgtgttgctgatgagtcagctgaaaattgtgacaaatcacttcataccctttttggagacaaattatgcacagttgcaactcttcgtgaaacctatggtgaaatggctgactgctgtgcaaaacaagaacctgagagaaatgaatgcttcttgcaacacaaagatgacaacccaaacctcccccgattggtgagaccagaggttgatgtgatgtgcactgcttttcatgacaatgaagagacatttttgaaaaaatacttatatgaaattgccagaagacatccttacttttatgccccggaactccttttctttgctaaaaggtataaagctgcttttacagaatgttgccaagctgctgataaagctgcctgcctgttgccaaagctcgatgaacttcgggatgaagggaaggcttcgtctgccaaacagagactcaagtgtgccagtctccaaaaatttggagaaagagctttcaaagcatgggcagtagctcgcctgagccagagatttcccaaagctgagtttgcagaagtttccaagttagtgacagatcttaccaaagtccacacggaatgctgccatggagatctgcttgaatgtgctgatgacagggcggaccttgccaagtatatctgtgaaaatcaagattcgatctccagtaaactgaaggaatgctgtgaaaaacctctgttggaaaaatcccactgcattgccgaagtggaaaatgatgagatgcctgctgacttgccttcattagctgctgattttgttgaaagtaaggatgtttgcaaaaactatgctgaggcaaaggatgtcttcctgggcatgtttttgtatgaatatgcaagaaggcatcctgattactctgtcgtgctgctgctgagacttgccaagacatatgaaaccactctagagaagtgctgtgccgctgcagatcctcatgaatgctatgccaaagtgttcgatgaatttaaacctcttgtggaagagcctcagaatttaatcaaacaaaattgtgagctttttgagcagcttggagagtacaaattccagaatgcgctattagttcgttacaccaagaaagtaccccaagtgtcaactccaactcttgtagaggtctcaagaaacctaggaaaagtgggcagcaaatgttgtaaacatcctgaagcaaaaagaatgccctgtgcagaagactatctatccgtggtcctgaaccagttatgtgtgttgcatgagaaaacgccagtaagtgacagagtcaccaaatgctgcacagaatccttggtgaacaggcgaccatgcttttcagctctggaagtcgatgaaacatacgttcccaaagagtttaatgctgaaacattcaccttccatgcagatatatgcacactttctgagaaggagagacaaatcaagaaacaaactgcacttgttgagctcgtgaaacacaagcccaaggcaacaaaagagcaactgaaagctgttatggatgatttcgcagcttttgtagagaagtgctgcaaggctgacgataaggagacctgctttgccgaggagggtaaaaaacttgttgctgcaagtcaagctgccttaggcttataa

Protein sequence

>Seq1

MAGCKNFFWKTFTSCAGCKNFFWKTFTSCDAHKSEVAHRFKDLGEENFKALVLIAFAQYLQQCPFEDHVKLVNEVTEFAKTCVADESAENCDKSLHTLFGDKLCTVATLRETYGEMADCCAKQEPERNECFLQHKDDNPNLPRLVRPEVDVMCTAFHDNEETFLKKYLYEIARRHPYFYAPELLFFAKRYKAAFTECCQAADKAACLLPKLDELRDEGKASSAKQRLKCASLQKFGERAFKAWAVARLSQRFPKAEFAEVSKLVTDLTKVHTECCHGDLLECADDRADLAKYICENQDSISSKLKECCEKPLLEKSHCIAEVENDEMPADLPSLAADFVESKDVCKNYAEAKDVFLGMFLYEYARRHPDYSVVLLLRLAKTYETTLEKCCAAADPHECYAKVFDEFKPLVEEPQNLIKQNCELFEQLGEYKFQNALLVRYTKKVPQVSTPTLVEVSRNLGKVGSKCCKHPEAKRMPCAEDYLSVVLNQLCVLHEKTPVSDRVTKCCTESLVNRRPCFSALEVDETYVPKEFNAETFTFHADICTLSEKERQIKKQTALVELVKHKPKATKEQLKAVMDDFAAFVEKCCKADDKETCFAEEGKKLVAASQAALGL *
