# Supplementary material for: Combined anti-tumor efficacy of somatostatin fusion protein and vaccinia virus on tumor cells with high expression of somatostatin receptors
Source: Sci Rep. 2022 Oct 7;12:16885. doi: 10.1038/s41598-022-21506-8 (PMC9547013; doi:10.1038/s41598-022-21506-8)
Supplement: Supplementary file 2 — Supplementary Information 2. [file 41598_2022_21506_MOESM2_ESM.pdf]

Fig. 1

$(sst14)_2$-HSA   anti-HSA   70kDa

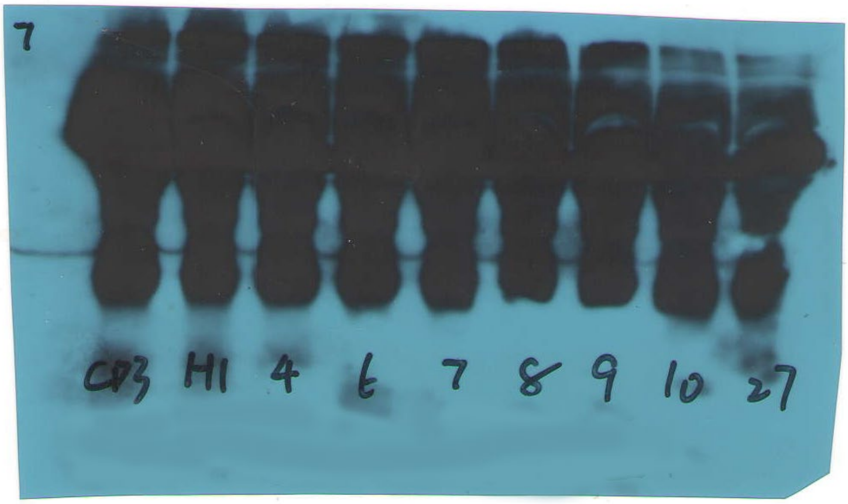

$(sst14)_2$-HSA   anti-somatostatin   70kDa

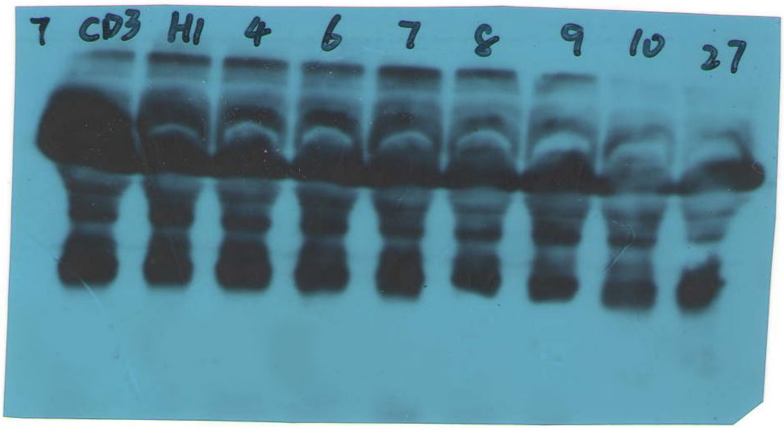

Actin   43kDa

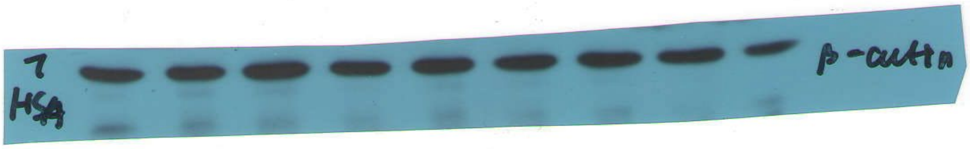

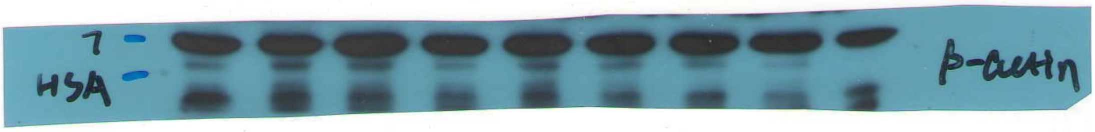

Fig. 2

SSTR1 65kDa (43 kDa)

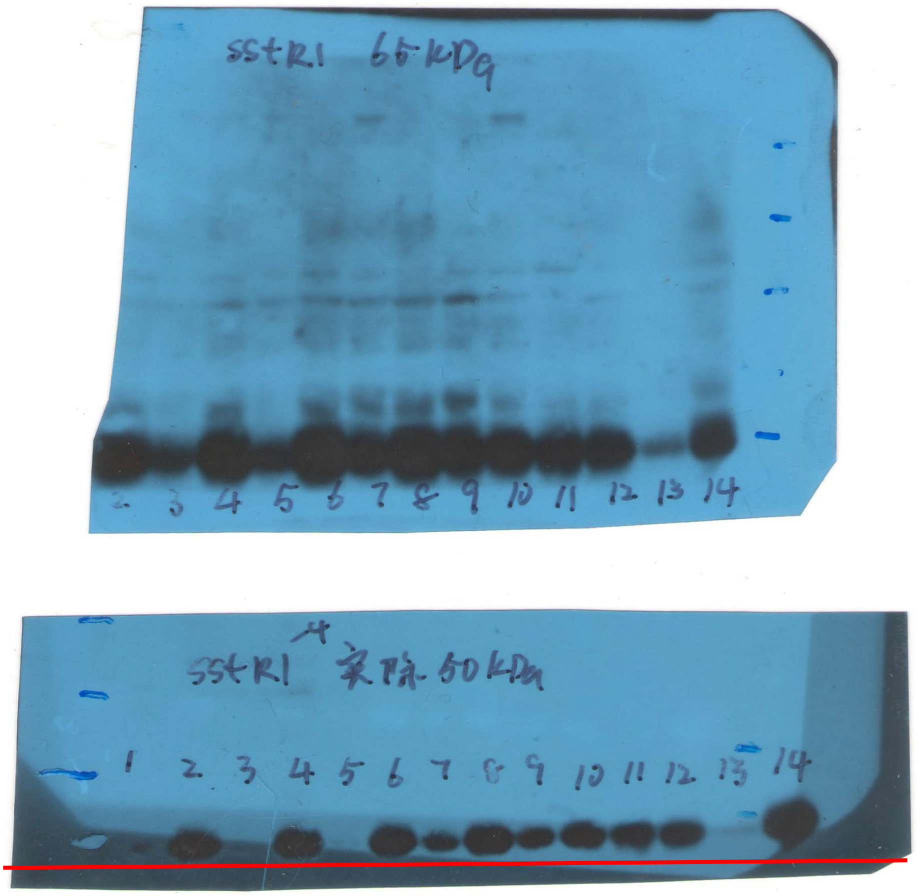

SSTR2 (41 kDa)

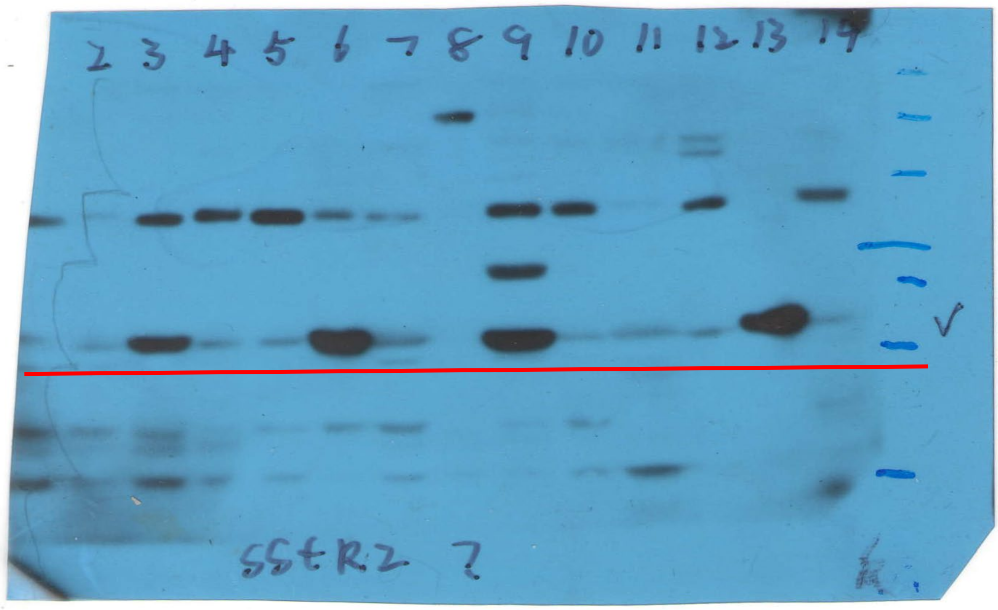

SSTR3 (46 kDa)

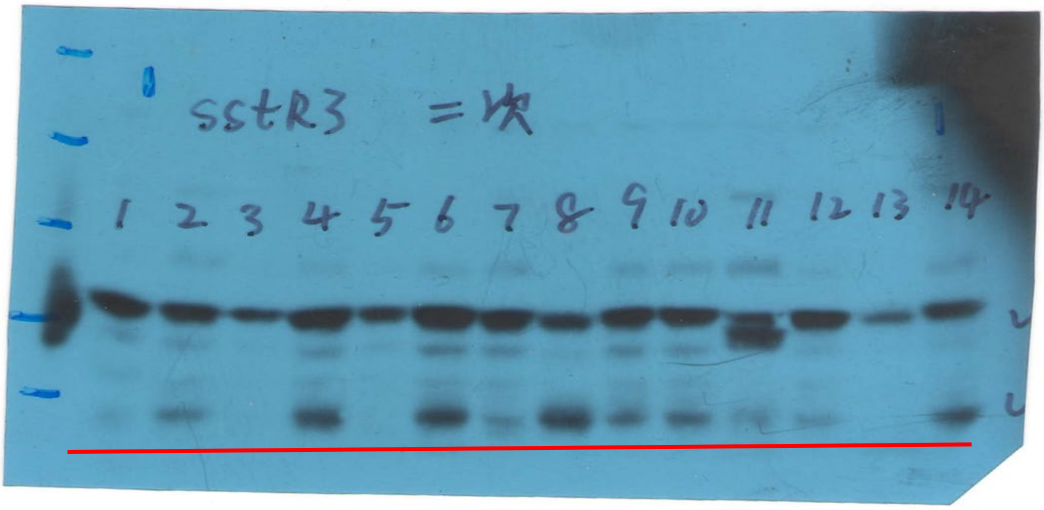

SSTR4 70kDa (43 kDa)

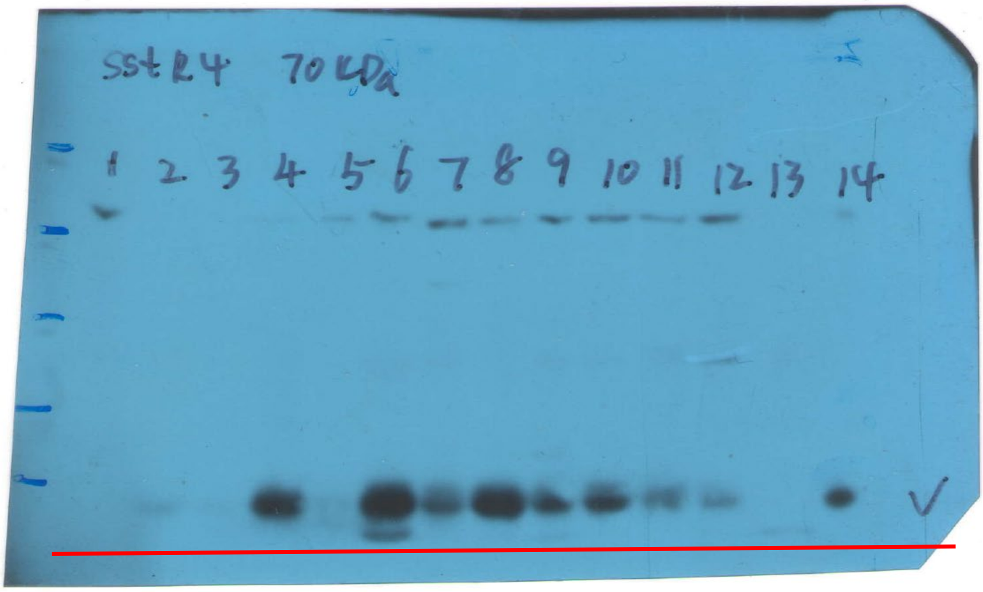

SSTR5 39/74kDa (40 kDa)

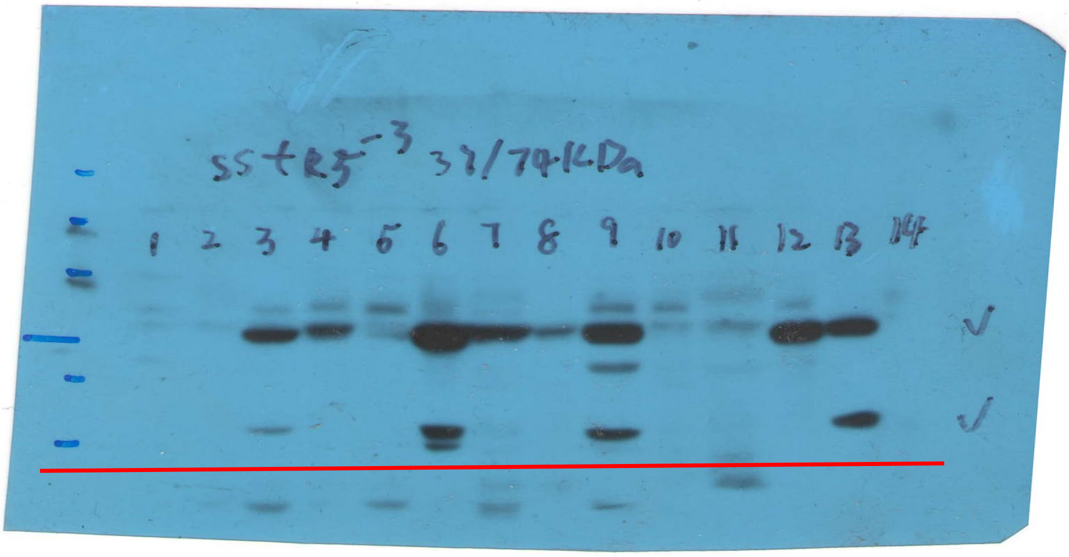

Tubulin 55kDa

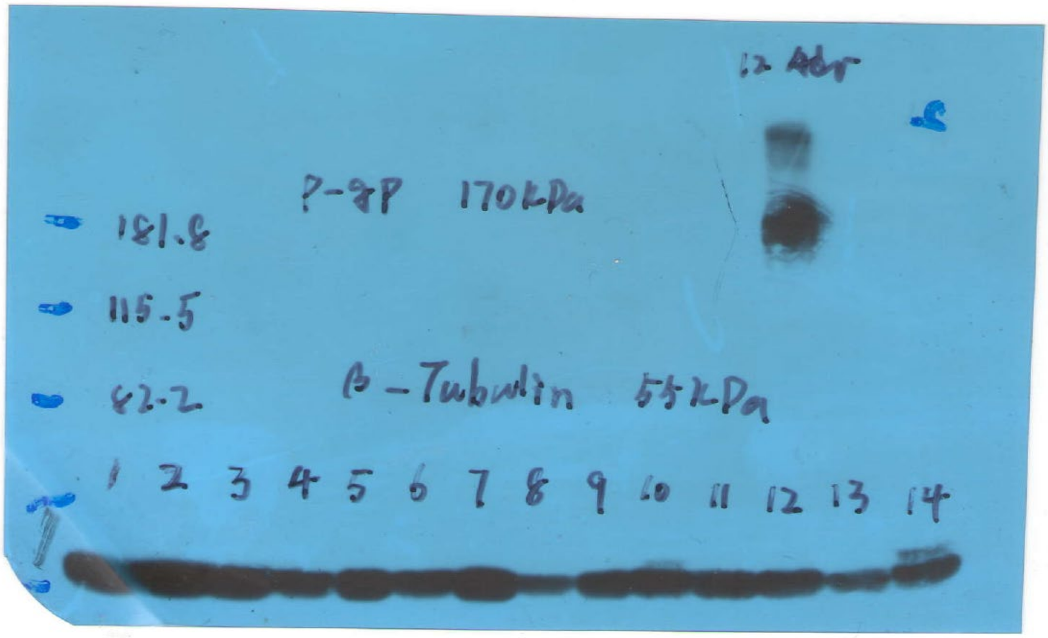

Predicted band size

SSTR1	618-1793bp	391Aa*110=43kDa
SSTR2	361-1470bp	369Aa*110=40.6kDa
SSTR3	526-1782bp	418Aa*110=46kDa
SSTR4	65-1231bp	388Aa*110=42.7kDa
SSTR5	89-1183bp	364Aa*110=40kDa
